# Mutation in E1, the Ubiquitin Activating Enzyme, Reduces *Drosophila* Lifespan and Results in Motor Impairment

**DOI:** 10.1371/journal.pone.0032835

**Published:** 2013-01-29

**Authors:** Hsiu-Yu Liu, Cathie M. Pfleger

**Affiliations:** 1 Department of Oncological Sciences, The Mount Sinai School of Medicine, New York, New York, United States of America; Brigham and Women’s Hospital, Harvard Medical School, United States of America

## Abstract

Neurodegenerative diseases cause tremendous suffering for those afflicted and their families. Many of these diseases involve accumulation of mis-folded or aggregated proteins thought to play a causal role in disease pathology. Ubiquitinated proteins are often found in these protein aggregates, and the aggregates themselves have been shown to inhibit the activity of the proteasome. These and other alterations in the Ubiquitin Pathway observed in neurodegenerative diseases have led to the question of whether impairment of the Ubiquitin Pathway on its own can increase mortality or if ongoing neurodegeneration alters Ubiquitin Pathway function as a side-effect. To address the role of the Ubiquitin Pathway *in vivo*, we studied loss-of-function mutations in the *Drosophila* Ubiquitin Activating Enzyme, *Uba1* or E1, the most upstream enzyme in the Ubiquitin Pathway. Loss of only one functional copy of E1 caused a significant reduction in adult lifespan. Rare homozygous hypomorphic E1 mutants reached adulthood. These mutants exhibited further reduced lifespan and showed inappropriate Ras activation in the brain. Removing just one functional copy of Ras restored the lifespan of heterozygous E1 mutants to that of wild-type flies and increased the survival of homozygous E1 mutants. E1 homozygous mutants also showed severe motor impairment. Our findings suggest that processes that impair the Ubiquitin Pathway are sufficient to cause early mortality. Reduced lifespan and motor impairment are seen in the human disease X-linked Infantile Spinal Muscular Atrophy, which is associated with mutation in human E1 warranting further analysis of these mutants as a potential animal model for study of this disease.

## Introduction

### Aggregation Prone Neurodegenerative Diseases

Neurodegenerative diseases are a major cause of mortality and can cause a range of devastating symptoms. While these diseases have a number of symptomatic differences, they also share key features that could reflect a common underlying pathology. For example, aggregated proteins are found in the brains of patients in many of these diseases, and it is currently believed that these aggregates play an important role in pathology of the diseases [1]–[21]. Currently, at least 4.5 million people in the United States, roughly 1 in 68, suffer from Alzheimer’s Disease (AD), the most common form of dementia, and prevalence of this disease increases exponentially with advancing age and afflicts one third to one half of all people over age 85 [22]–[23]. In AD, a number of proteins have been shown to adopt abnormal conformations and/or to aggregate. For example, the microtubule-associated protein tau adopts abnormal conformations forming neurofibrillary tangles (NFT), a typical feature of AD and taupoathies [3]–[6]. In addition, inappropriate processing of amyloid-beta (Aß) results in Aß peptides, which form extracellular plaques [6]–[8]. Parkinson’s Disease (PD), another condition with increasing incidence upon aging, is the second most common cause of dementia [9]–[10], [24]–[26]. Pathology in PD is thought in part to result from aggregation of the protein alpha-synuclein. Given the large population now entering the relevant ages for typical diagnosis, the number of people afflicted with AD and PD will climb dramatically in coming decades. 1 in 10,000 people suffers from Huntington’s Disease (HD), a dominant neurodegenerative condition. HD results from expansion of the polyglutamine (polyQ) repeats of the gene *huntingtin* (*htt*); polyQ-expanded forms of the *htt* protein form protein aggregates [11], [27]. Expansion of polyQ stretches are also implicated in other neurodegenerative diseases [20]–[21].

### The Ubiquitin Pathway and Neurodegenerative Diseases

One of the major pathways responsible for clearing mis-folded or aggregated proteins from a cell is the Ubiquitin Pathway. The Ubiquitin Pathway consists of a series of enzymes responsible for attaching the small protein ubiquitin to substrate proteins. In the most upstream step, a Ubiquitin Activating Enzyme, E1, charges ubiquitin and transfers ubiquitin to a Ubiquitin Conjugating Enzyme, E2. The E2 then transfers ubiquitin to a Ubiquitin Protein ligase, E3, or works with an E3 to conjugate ubiquitin to a substrate protein. Ubiquitin can be conjugated to a substrate singly or in a poly-ubiquitin chain. Once ubiquitinated, substrates are then directed to a variety of potential fates including endocytosis and degradation [28]–[31]. Normally, mis-folded or aggregated proteins can be poly-ubiquitinated and then degraded by the 26S proteasome [1]–[2], [32]–[35].

In AD, PD, and HD, ubiquitinated proteins have been shown to accumulate in inclusions and in protein aggregates. Moreover, isolated Aß_1–42_ aggregates, tau aggregates, alpha synuclein aggregates, and polyQ aggregates have been shown to inhibit proteasome function *in vitro* [13]–[19]. Other findings have also implicated the Ubiquitin Pathway in neurodegenerative diseases. For example, a reduced level of E1 has been found in the cytosol of AD patients [32], and one of the familial forms of PD is caused by mutation in a gene called *parkin* that encodes an E3 enzyme [33]–[35]. Some AD patients also show the presence of, UBB+1, a frameshift mutant of ubiquitin that can inhibit the proteasome once it accumulates in a cell but which cannot be attached to substrate proteins to target them for degradation [36]–[42].

These findings together raise the question of whether impairment of the Ubiquitin Pathway on its own can promote increased mortality as a general mechanism underlying a broad spectrum of neurodegenerative diseases or if impairment of the Ubiquitin Pathway occurs largely as a side-effect in neurodegenerative processes.

### *Drosophila* Models of Age-related Diseases

Many crucial signaling pathways and important processes are conserved between *Drosophila* and humans. In fact, more than 70% of genes associated with human diseases have *Drosophlia* sequence homologs [43]. Because *in vivo* assays can address growth, proliferation, apoptosis, and longevity in *Drosophila,* this system confers the ability to address the functional relevance of genes to disease-associated phenotypes by genetic manipulation. Thus, *Drosophila* can make substantial contributions to understanding human diseases.

We examined lifespan in different genetic backgrounds in *Drosophila*. Our findings suggest that impairing the Ubiquitin Pathway is sufficient to promote early mortality. We report here that mutation in one or both copies of *Drosophila* E1 on its own promoted a dramatic reduction in lifespan. Flies carrying two mutant copies of E1 also demonstrated dramatic motor impairment and aberrant Ras signaling in adult brains. Importantly, the reduced lifespan associated with mutation in one copy of E1 was completely suppressed by reducing the gene dosage of Ras while the reduced lifespan resulting from mutation in both copies of E1 was partially suppressed by reducing the gene dosage of Ras.

## Results

Impairment of the Ubiquitin Pathway is implicated in normal aging and in a number of neurodegenerative diseases including AD, HD, and PD. In order to evaluate how loss of ubiquitination could affect lifespan using an *in vivo* model, we utilized loss-of-function mutations in E1, the Ubiquitin Activating Enzyme that we isolated previously [44]–[45]. E1 is the most upstream enzyme in the pathway and has no specificity for downstream targets. Therefore, loss of E1 is expected to affect all downstream steps. Moreover, a number of variants have been reported in the human E1 gene, *Ube1*, including confirmed loss-of-function alleles [46]. To avoid confusion, both human *Ube1* and *Drosophila Uba1* will be referred to hereafter as simply E1, and *Drosophila* mutant alleles will be referred to with allele-specific designations (*Uba1^B1^*, *Uba1^B2^*, *Uba1^A1^*, *Uba1^A3^*, and *Uba1^A5^*) as appropriate.

**Figure 1:**
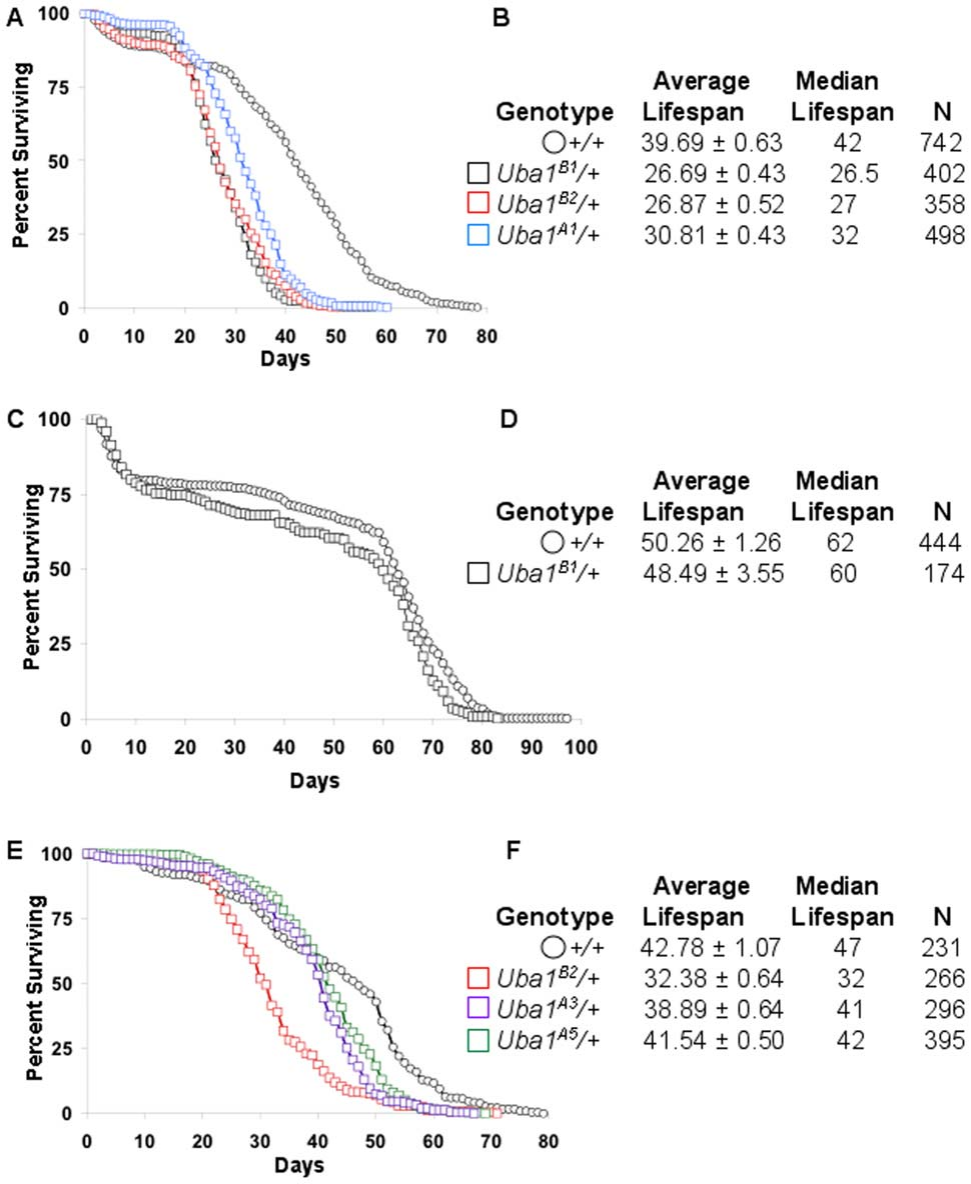
Mutation in E1, the Ubiquitin Activating Enzyme, dominantly reduces lifespan. (A, C, E) Graphs showing the percentage of (A, E) male and (C) female flies surviving versus time in days for wild-type +/+ control flies (open circles, black A, C, E) and for flies carrying one mutant copy of E1 (open squares). E1 heterozygous mutants tested were *Uba1^B1^/+* (black open squares, A, C), *Uba1^B2^/+* (red open squares, A, E), *Uba1^A1^/+* (blue open squares, A), *Uba1^A3^/+* (purple open squares, E), *Uba1^A5^/+* (green open squares, E). (B, D, F) Tables summarizing the average ± s.e.m. and median lifespan and the number of flies followed (N) for each genotype graphed in (A, C, E) respectively. Detailed genotypes for flies in this and subsequent figures are detailed in the Materials and Methods section.

Removing one copy of a gene often has no obvious effect because the remaining wild-type copy can allow for production of sufficient levels of the gene product. In some cases, however, loss of one copy of a gene can result in limiting levels of that gene product and can result in attenuation of downstream processes. Therefore, we examined flies carrying one mutant copy of E1. Flies heterozygous for mutation in E1 show no visible abnormalities when compared to wild-type flies (not shown). These flies emerge from their pupal cases at the expected Mendelian frequencies and are fertile. Despite the lack of a visible phenotype, heterozygous mutations can create sensitized genetic backgrounds or even promote disease symptoms on their own. We examined the lifespan of flies carrying a mutant copy of E1. In parallel assays, flies carrying just one mutant copy of E1 showed a dramatic decrease in lifespan compared to wild-type control flies (Fig. 1A–D). Control male flies lived an average of 39.69±0.63 days, whereas *Uba1^B1^*/+ male flies lived an average of only 26.69±0.43 days, *Uba1^B2^*/+ male flies lived only 26.87±0.52 days, and *Uba1^A1^*/+ male flies lived an average of 30.81±0.43 days. In all three cases, Kaplan-Meier survival curve statistical analysis indicated that the reduced survival was extremely significantly different from control flies (P<0.0001). Mated females demonstrate a shortened lifespan compared to virgin females due to the Sex peptide [47]–[48]. Therefore we did not examine mated females. Virgin females carrying one mutant copy of E1 also showed a statistically significant decline in lifespan compared to controls (P = 0.0041 by log rank Mantel-Cox, P = 0.0293 by Gehan-Breslow-Wilcoxon), although this decline was less dramatic than in males (shown for *Uba1^B1^* in Fig. 1C–D).

Surprisingly, male flies carrying the E1 null allele *Uba1^A1^* (which produces a truncated form of the protein [44]) showed statistically significant increased survival compared to flies heterozygous for the hypomorphic mutations *Uba1^B1^* and *Uba1^B2^*. Therefore, we examined additional null mutations in E1, alleles *Uba1^A3^* and *Uba1^A5^* each of which produces full-length protein that lacks activity [44]. Similar to *Uba1^A1^*, male flies heterozygous for either *Uba1^A3^* or *Uba1^A5^* showed a statistically significant difference in survival compared to male flies heterozygous for the hypomorphic mutation *Uba1^B2^* (Fig. 1E–F). A number of possibilities could underlie this phenomenon. Because E1 function is essential at a cellular level, perhaps a feedback mechanism senses overall levels of activity to promote increased E1 expression if levels fall short of a critical threshold. Such a feedback mechanism could be activated in flies heterozygous for a null mutation (where the level falls short) but not in flies heterozygous for hypomorphic mutation (presumably the level does not fall short). Alternatively, the null alleles may fail to interact with other pathway components, but if the hypomorphic proteins interact less productively with binding partners, they could thereby sequester such components from the wild-type protein, resulting in reduced pathway activity.

Motor function in *Drosophila* can be assessed using standard “negative geotaxis” climbing assays [49]–[50]. When tapped to the bottom of a vial, wild-type flies immediately climb towards the top of the vial. In contrast, flies suffering from motor problems cannot easily climb to the same height as wild-type flies in a similar amount of time. We assessed motor function in E1 mutants by counting the percentage of flies capable of climbing to a height of 4 cm within 5 seconds. Despite the decline in lifespan, heterozygous mutation in E1 did not significantly impair motor function (data not shown) when compared to age-matched control flies.

### E1 Homozygous Mutants Often Show Patterning Abnormalities

We reported previously that flies homozygous for null mutations in E1 are embryonic lethal, while flies homozygous for hypomorphic mutations have dramatically reduced viability [45]. As described, flies carrying only one mutant copy of E1 have no visible phenotypes although they show a significant reduction in lifespan. In contrast, flies carrying two hypomorphic mutant copies of E1 have visible and severe phenotypes that may result from processes dysregulated in these mutants during development. Importantly, these abnormalities could reveal insights into the mechanisms underlying the mutant phenotypes observed for mutation in one or both copies of E1.

**Figure 2:**
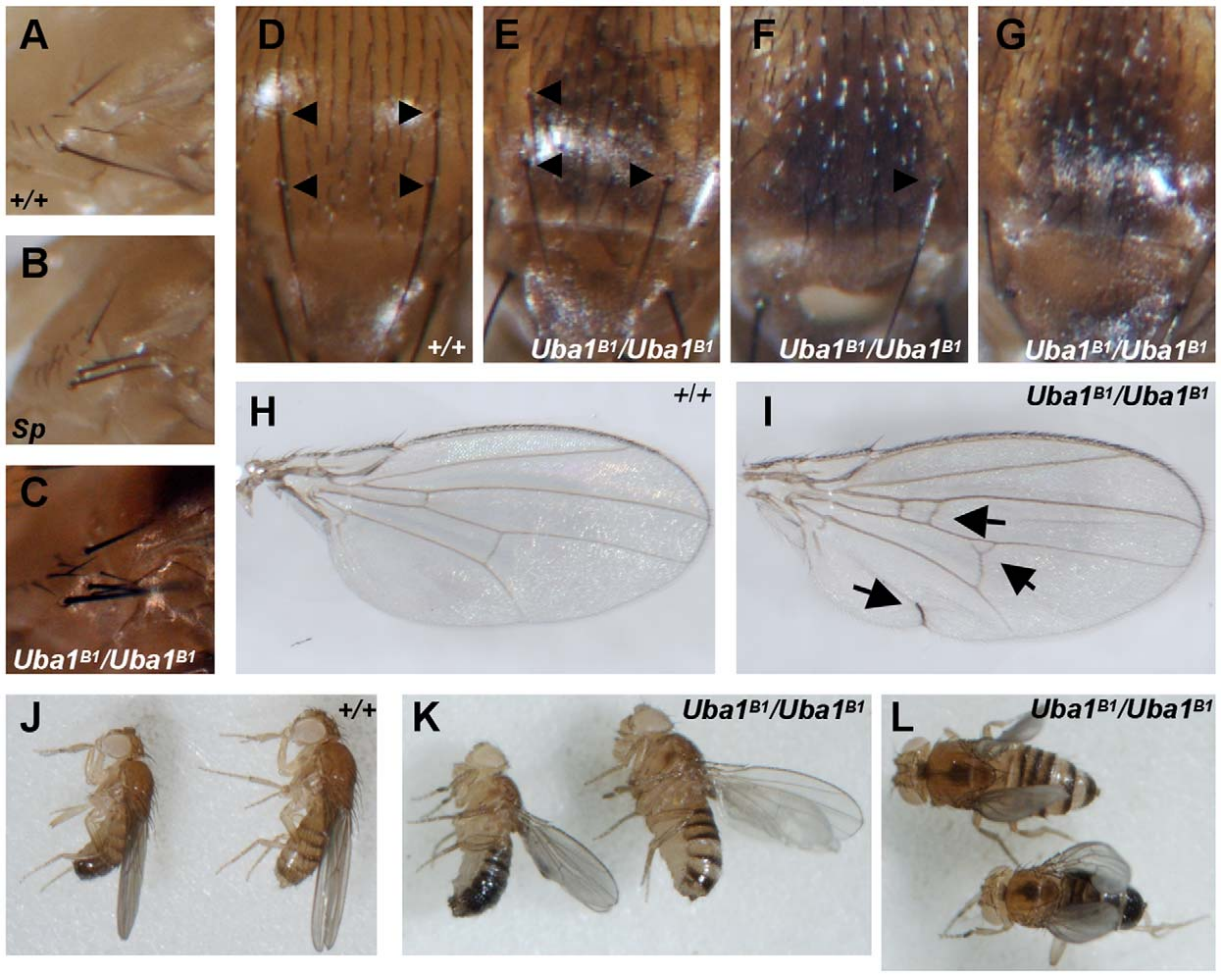
*Uba1^B1^* homozygotes have patterning abnormalities. Flies homozygous for the E1 mutation *Uba1^B1^* have a number of patterning abnormalities. (A) The normal pattern of sternopleural bristles is shown for a wild-type fly. (B) The dominant marker *Sternopleural (Sp)*, alters the pattern of sternopleural bristles to cause an increase in the number of bristles. (C) *Uba1^B1^/Uba1^B1^* flies have extra sternopleural bristles. (D) In a wild-type fly, there are four large dorsal mechanosensory bristles (arrows in D–G). (E–G) *Uba1^B1^/Uba1^B1^* flies frequently show loss of one (E) or more (F–G) of these bristles. (H) The normal pattern of wing veination is shown for a wild-type control fly. (I) *Uba1^B1^/Uba1^B1^* flies frequently show wing abnormalities including extra wing vein material (arrows). Female wings are shown. (J) Normal wing posture is shown for wild-type flies (an example of a male to the left, female to the right). (K–L) *Uba1^B1^/Uba1^B1^* flies show abnormal wing posture; wings are typically held out from the body at an odd angle, and are sometimes turned downward. (K) Side view and (L) overhead view of the same mutants. Male to the left in (K) and bottom in (L); female to the right in (K) and top in (L).

Flies homozygous for the hypomorphic mutation *Uba1^B2^* die during late larval or pupal stages and do not reach adulthood. Very few *Uba1^B1^*/*Uba1^B1^* flies or *Uba1^B1^*/*Uba1^B2^* flies reach adulthood. Those mutant flies that do reach adulthood typically display a number of obvious abnormalities including rare outgrowths [45], and they appear to be infertile. We report here additional abnormalities in the wing as well as mis-patterned bristles including disruption of the bristle patterns on the notum and in the sternopleural region. *Uba1^B1^*/*Uba1^B1^* flies frequently exhibit extra sternopleural bristles (Fig. 2C) compared to wild-type flies (Fig. 2A) that resemble the *Sternopleural (Sp)* mutant phenotype (Fig. 2B). Extra sternopleural bristles sometimes form as a consequence from local increased *wingless* signaling [51]. This would be consistent with our previous findings of *wingless* accumulation in E1 null mutant clones in both the wing and the eye in regions of *wingless* expression [44]. We also frequently observe loss of one or more dorsocentral mechanosensory bristles (Fig. 2E–G) compared to the wild-type pattern (Fig. 2D).

We reported previously that *Uba1^B1^*/*Uba1^B1^* and *Uba1^B2^*/*Uba1^B2^* larvae display a dramatic increase in Ras signaling through ERK [45]. Ras signaling is involved in specification of the wing vein, and increased Ras signaling can promote formation of ectopic wing vein material [52]–[53]. Consistent with this, *Uba1^B1^*/*Uba1^B1^* and *Uba1^B1^*/*Uba1^B2^* adult wings often display extra wing vein material (Fig. 2I) compared to wild-type wings (Fig. 2H).

### E1 Mutants Demonstrate Motor Impairment

**Figure 3:**
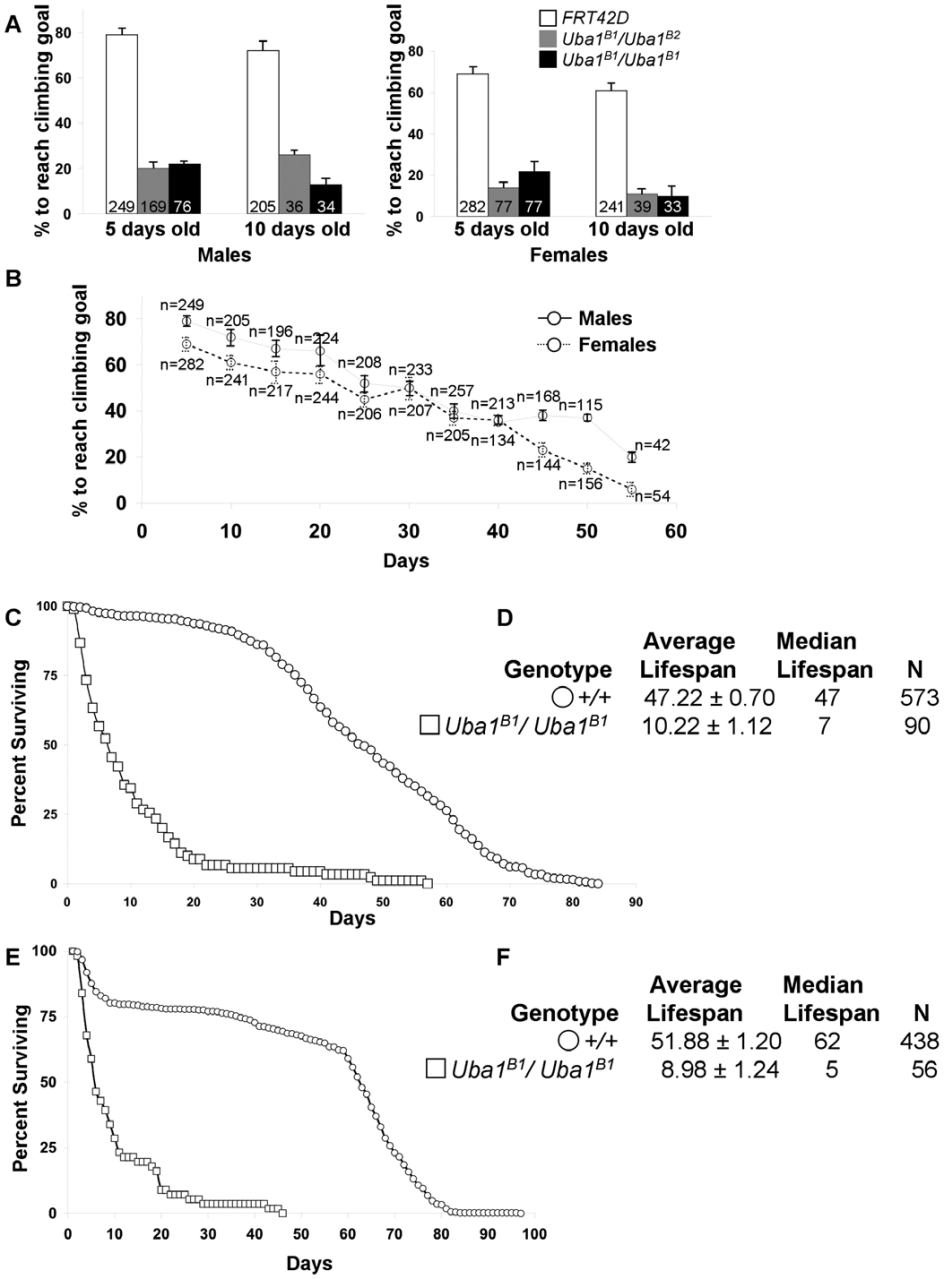
E1 homozygous mutant flies exhibit dramatic motor impairment and reduced lifespan. (A) *Uba1^B1^/Uba1^B1^* homozygous flies and *Uba1^B1^/Uba1^B2^* flies demonstrate a dramatic reduction in the ability to climb compared to wild-type +/+ flies. Flies were tested for the ability to climb 4 cm in 5 seconds. Trials of age-matched flies were tested 5 times in small groups of typically 10–15 flies. The age, gender, and total number of flies tested for each genotype is indicated. (B) Climbing assays for wild-type *+/+* flies for flies aged 5 days to 55 days. Both males (solid line) and females (dashed line) were tested. The total number of flies tested for each gender and time point is indicated above each data point. Standard Deviation for each data point based on five replicate trials for each genotype is indicated by error bars for A–B. (C, E) Graph showing the percentage of (C) male or (E) female flies surviving versus time in days for wild-type (+/+, open circles) control flies or *Uba1^B1^/Uba1^B1^* homozygous flies (open squares) over time in days. *Uba1^B1^/Uba1^B1^* homozygous flies show a dramatic decrease in lifespan compared to wild-type controls. (D, F) Table summarizing the average ± s.e.m. and median lifespans and the number of flies followed (N) for each of the genotypes graphed in (C, F) respectively.

The wings of *Uba1^B1^*/*Uba1^B1^* flies are typically held out at an abnormal angle (Fig. 2K–L) compared to wild-type posture (Fig. 2J). Such wing posture can reflect problems with muscles or motor neurons [54]–[55], so we investigated the motor function of these *Uba1^B1^/Uba1^B1^* and *Uba1^B1^/Uba1^B2^* mutants. 5 days after emerging from their pupal cases, 79 percent of males flies and 69 percent of female flies of the control genotype *w; FRT42D* are capable climbing 4 cm in 5 seconds. In contrast, only about 20 percent of 5 day-old and 10 day-old *Uba1^B1^/Uba1^B1^* and *Uba1^B1^/Uba1^B2^* E1 mutants can do so (Fig. 3A). Flies were tested in groups, not individually; therefore, climbing ability reflects the motor function in the population. While there appears to be an increase in motor function in *Uba1^B1^/Uba1^B2^* males from 5 days to 10 days of age, because we tested each genotype as a population and did not track individuals, this increase may reflect that those flies surviving to 10 days were healthier overall than their siblings who were tested at 5 days but did not live until 10 days.

Motor function declines with age in flies just as it does in humans. Control *FRT42D* flies were tested in climbing assays every 5 days. Both male and flies showed a gradual decline in climbing ability over time (Fig. 3B). Interestingly, the motor impairment of the *Uba1^B1^*/*Uba1^B1^* and *Uba1^B1^*/*Uba1^B2^* mutants resembled that of control flies of extremely advanced age. 50 day-old female control flies and 55 day-old male control flies showed a motor performance similar to that of 5 and 10 day-old *Uba1^B1^*/*Uba1^B1^* and *Uba1^B1^*/*Uba1^B2^* flies.

### E1 Mutants Exhibit Dramatically Reduced Survival

In addition to their reduced survival to adulthood, E1 mutants that reached adulthood were extremely short-lived. In parallel assays, wild-type *w; FRT42D* control male flies lived 47.22±0.70 days on average, whereas *Uba1^B1^*/*Uba1^B1^* male mutants lived only an average of 10.22±1.12 days (Fig. 3C–D). In a separate trial, wild-type *w; FRT42D* control female virgin flies lived 51.88±1.20 days on average, and *Uba1^B1^*/*Uba1^B1^* female virgins lived only an average of 8.98±1.24 days (Fig. 3E–F). In both males and females, the reduction in lifespan was extremely statistically significant (P<0.0001).

### Inappropriate Ras Upregulation in the Brains of E1 Mutants

**Figure 4:**
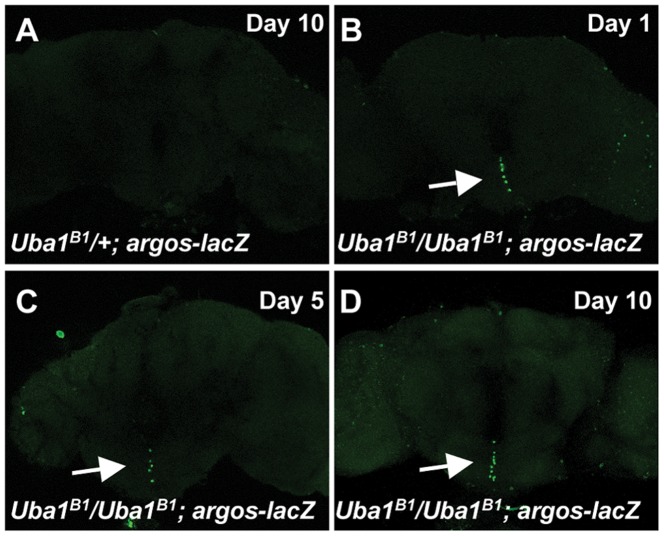
Inappropriate Ras activation in the brains of E1 homozygous mutant. *Argos* is a high threshold target of Ras signaling. Expression of *argos* can be monitored using an *argos-lacZ* reporter. Upon high Ras activation, *lacZ* is transcribed in the pattern of *argos* and the gene product can be detected using immunohistochemical methods with antibodies to β-gal. (A) In flies carrying the *argos-lacZ* reporter and mutant for only one copy of E1 (genotype *Uba1^B1^/+; aos-lacZ/+)*, no significant *argos* expression (green) is detected. Brains from flies aged 1 day, 5 days, and 10 days were examined. Control brain shown is a brain from a 10 day-old fly. (B–D) In contrast, in the brains of flies homozygous for mutation in E1 with the *argos-lacZ* reporter, (*Uba1^B1^/Uba1^B1^*; *aos-lacZ/+*), a number of cells show strong *argos* expression in mutant brains (green, arrow). Shown are brains dissected from flies at 1 day old (B), 5 days old (C), and 10 days old (D).

We showed previously that *Uba1^B1^*/*Uba1^B1^* and *Uba1^B2^*/*Uba1^B2^* mutant larvae exhibit an increase in Ras signaling through ERK [45]. In *Drosophila,* Ras activation results in expression of Ras target genes including the high-threshold target *argos*. Using an *argos-lacZ* reporter, we investigated if Ras signaling is altered in *Uba1^B1^*/*Uba1^B1^* adult brains. At 1 day, 5 days, and 10 days of age, there is no obvious *argos* expression in the brains of *Uba1^B1^*/+ flies (Fig. 4A, shown for a 10 day old brain). In contrast, brains dissected from 1 day-old, 5 day-old, and 10-day old *Uba1^B1^*/*Uba1^B1^* mutant flies show a number of cells with clear *argos* expression (Fig. 4B–D) indicating inappropriate Ras activation.

We attempted to examine these brains for caspase activation by staining with antibodies to the activated form of caspase 3 (anti-C3). We did not observe an obvious increase in anti-C3 staining in E1 homozygous mutant brains compared to age-matched control brains (data not shown). If cell death occurred gradually over time, it would be difficult to visualize by analysis of individual time points, and a massive wave of cell death could be missed by examining the wrong time points. Alternatively, it is possible that cell death occurred in a caspase-independent fashion or that the reduction in lifespan did not involve an increase in cell death in these brains.

### The Reduced Survival of E1 Mutants is Sensitive to the Gene Dosage of Ras

**Figure 5:**
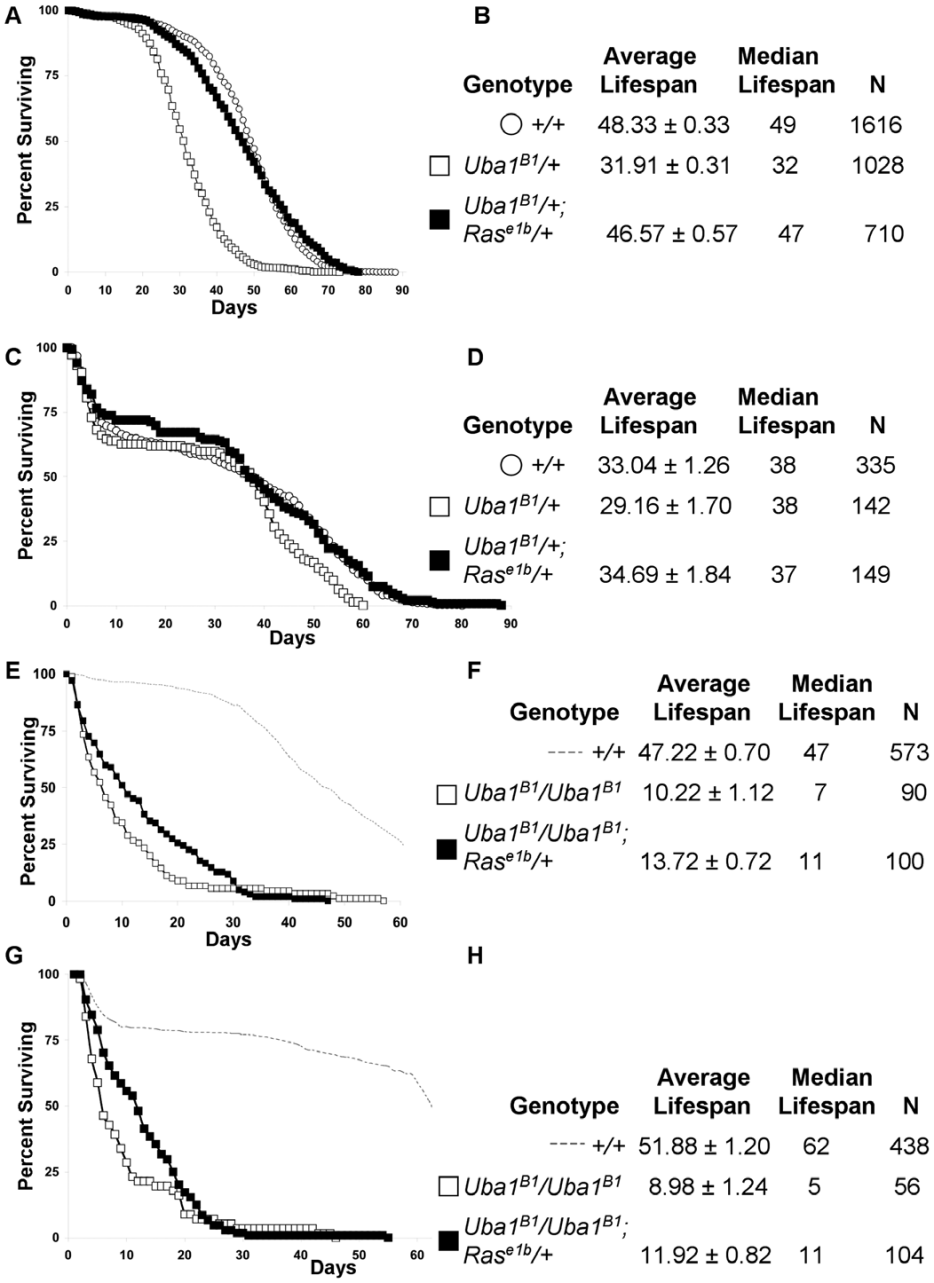
Ras mutation dominantly rescues the reduced lifespan resulting from heterozygous or homozygous mutation in E1. (A, C) Graphs showing the percentage of (A) male and (C) female flies surviving versus time in days for wild-type (+/+) control flies (open circles), for flies carrying one mutant copy of E1 (*Uba1^B1^/+*, open squares), and for flies carrying one mutant copy of E1 and one mutant copy of Ras (*Uba1^B1^/+; Ras^e1b^/+*, filled in squares). Mutation in just one copy of Ras restored the lifespan of E1 heterozygous mutants to that of wild-type controls. (B, D) Tables summarizing the average ± s.e.m. and median lifespans for each of the genotypes graphed in (A, C) respectively and the number of flies (N) followed for each genotype. For the trial shown in (C–D), control flies showed decreased survival compared to other trials; this affected all genotypes and may reflect a difference in food or environment. Despite this, the effects of genotype remained consistent. (E, G) Graph showing the percentage of (E) male and (G) female flies surviving versus time in days for E1 homozygous mutant flies (*Uba1^B1^/Uba1^B1^*, open squares) or E1 homozygous mutant flies carrying one mutant copy of Ras (*Uba1^B1^/Uba1^B1^; Ras^e1b^/+*, filled in squares). Mutation in Ras increases the survival of the homozygous E1 mutants. (F, H) Tables indicating average ± s.e.m. and median lifespan for each of the genotypes in the experiments in (E, G) respectively and the number of flies (N) followed.

Increased Ras activation can promote AD-like changes in neuronal cells in culture [56], and we previously found that the reduced survival to adulthood of E1 homozygous mutants was sensitive to the gene dosage of Ras [45]. We tested if mutation in Ras could rescue the mortality of *Uba1^B1^*/*+* and *Uba1^B1^*/*Uba1^B1^* mutant adult flies. Reducing the gene dosage of Ras by introducing one copy of the mutant allele *Ras^e1b^* significantly increased the lifespan of flies carrying one or two mutant copies of E1 (Fig. 5).

*Uba1^B1^*/+; *Ras^e1b^*/+ male flies had an average lifespan of 46.82 days, a statistically significant improvement (P<0.0001) from 31.91 days of *Uba1^B1^*/+ male flies in a parallel assay (Fig. 5A–B). In a separate trial, *Uba1^B1^*/+; *Ras^e1b^*/+ virgin females had an average lifespan of 34.69 days, statistically significantly improved (P = 0.0003 by Log-Rank Mantel-Cox, and P = 0.0379 by Behan-Breslow-Wilson) from 29.16 days of *Uba1^B1^*/+ virgin females (Fig. 5C–D). Under parallel conditions in each of these experiments, control male flies lived an average of 48 days and control female flies an average of 33.04 days. Importantly, the rescued survival of *Uba1^B1^*/+; *Ras^e1b^*/+ flies was not statistically significantly different from wild-type controls for both males and females. *Uba1^B1^*/*Uba1^B1^*; *Ras^e1b^*/+ males flies also showed a statistically significant (P<0.0001) increase in lifespan to 13.72 days from 10.22 days of *Uba1^B1^*/*Uba1^B1^* male flies (Fig. 5E–F); *Uba1^B1^*/*Uba1^B1^*; *Ras^e1b^*/+ virgin females showed a statistically significant (P<0.0001) increase in lifespan to 11.92 days from 8.98 days of *Uba1^B1^*/*Uba1^B1^* virgin females (Fig. 5G–H). The suppression of mortality by mutation in Ras in flies mutant in one or both copies of E1 in male and female flies suggests that Ras signaling likely plays a role in their reduced lifespan.

## Discussion

### Mutation in E1 as a Factor in Normal Age-related Decline and Age-related Neurodegenerative Diseases?

We have presented studies in *Drosophila* showing that loss-of-function mutations in only one copy of E1 have a dramatic effect on lifespan even in the absence of other mutations. In humans, E1 is encoded by the gene *Ube1* on the X chromosome. Given the high conservation of genes in the Ubiquitin Pathway, this could mean that women carrying one mutant copy of E1 might be at risk for reduced lifespan. How does loss of only one copy of E1 cause such a change in lifespan? The Ubiquitin Pathway controls a number of crucial cellular activities including signal transduction, apoptosis, and proteasome-mediated protein degradation. Proteasome activity and assembly decline with increased age [57]–[59]. Therefore, it is possible that at a young age, the threshold of E1 is easily met by only one functional genomic copy, but that as age advances and the proteasome becomes more limiting, that one copy of E1 is no longer sufficient to allow for clearance of misfolded or aggregating proteins. Thus, one possible explanation is that increased protein aggregation in flies with only one functional copy of E1 could cause increased mortality.

Disease-associated mutations in specific genes have been identified in familial forms of a number of neurodegenerative diseases including HD, AD, and PD as reviewed earlier. In HD, the length of the expanded polyQ region in part determines the age of onset of the disease; longer repeats often result in onset of symptoms at an earlier age. Intriguingly, however, patients with the same polyQ length do not always exhibit the same time of onset and course of the disease [60]–[62]. Therefore, polyQ length alone cannot explain all differences in disease presentation. Environmental factors and genetic background likely also contribute to variations in disease progression [63]–[64]. It will be exciting to explore if human E1 variants could create sensitive genetic backgrounds with adverse effects on the course of disease progression in patients suffering from HD. Moreover, there are familial cases of other neurodegenerative diseases in which causal mutations have not been identified. In addition, for some diseases, there are sporadic cases with no family history. In fact, sporadic AD is far more prevalent than familial AD, and the causes of sporadic AD also remain unclear [65]–[66]. Thus, it is highly likely that there are a number of genes whose mutation or dysregulation serve as risk factors or even causes of sporadic AD cases. We speculate that human E1 variants may serve as risk factors for the age-related decline in AD and other diseases. In the future, it will be important to address how loss of E1 affects lifespan in *Drosophila* neurodegeration models including models of HD and AD.

The Ubiquitin Pathway also regulates a number of signaling pathways including (but not limited to) Ras signaling. Upstream RTKs are down-regulated by ubiquitination [67]–[69], as is Ras itself [45], [70]. Therefore, another possibility is that upon aging, specific signaling pathways are dysregulated and contribute to reduced lifespan. In fact, examination of the brains of AD patients found evidence of increased Ras signaling [71]–[75]. Also, expressing activated Ras in neurons causes AD-type phenotypes in neurons in culture [56]. Importantly, we have shown here that reducing the gene dosage of Ras in flies carrying only one mutant copy of E1 restores lifespan to that of wild-type controls.

### A *Drosophila* Model for XL-SMA?

There are a number of variants reported for human E1 including loss-of-function alleles. In humans, the E1 gene *Ube1* is located on the X chromosome and has been lost from the Y chromosome [76], so a male inheriting a loss of function variant in E1 would have no wild-type copy. Some human E1 variants are associated with X-linked Infantile Spinal Muscular Atrophy (XL-SMA), a rare and severe form of Spinal Muscular Atrophy [46]. XL-SMA is a tragic condition in which males who inherit a mutant copy of E1 typically live less than two years and during which time they suffer terribly [46], [77]–[78]. Mothers who are carriers for a mutant copy of E1 often have a history of miscarriages presumably because many of their affected male children do not make it to term. XL- SMA has a similar presentation to the severe Type 1 SMA caused by mutation in the SMN1 gene, but also presents with congenital contractures [46], [77]–[78].

As we report here, flies homozygous for null mutations in E1 do not survive, but flies homozygous for hypomorphic E1 mutations can survive to adulthood at a very reduced rate, and these flies show a number of patterning abnormalities and severe motor impairment. Their lifespan is dramatically reduced compared to heterozygous mutants and wild-type controls.

To our knowledge, there is currently no animal model in which to study XL-SMA. We showed here that *Drosophila* E1 homozygous mutants recapitulate some aspects of human XL-SMA such as motor impairment and reduced lifespan. Thus, these *Drosophila* mutants warrant further study to determine if they recapitulate other aspects of this disease, such as degeneration of motorneurons reminiscent of the loss of anterior horn cells in XL-SMA, to establish if they could serve as an animal model to increase our understanding of this devastating disease. We previously showed that reducing the gene dosage of Ras in homozygous E1 mutants increases their survival to adulthood [45], and in this investigation we reported that it also extends their adult lifespan. If Ras signaling contributes to XL-SMA pathology in humans as it does to reduced lifespan in *Drosophila* E1 mutants, targeting Ras may serve as a potential therapeutic strategy for XL-SMA.

## Materials and Methods

### *Drosophila* Genotypes

Adult and larval images were from the following genotypes**:**

*w; FRT42D* (Figure 2A, 2D, 2H, 2J)

*Sp/CyO* (Figure 2B)

*w; FRT42D Uba1^B1^*/*FRT42D Uba1^B1^* (Figure 2C, 2E, 2F, 2G, 2I, 2K, 2L)

*w; FRT42D Uba1^B1^/+*; *aos-lacZ/+* (Figure 4A)

*w; FRT42D Uba1^B1^*/*FRT42D Uba1^B1^*; *aos-lacZ/+* (Figure 4B, 4C, 4D)

Adult genotypes in lifespan and motor assays were:

*W; FRT42D* (Figure 1A, 1C, 1E, 3B, 3E, 5A, 5C black open circles; Figure 3A, white bar; 5G gray dashed line)

*W; FRT42D Uba1^B1^*/*FRT42D* (Figure 1A,1C, black open squares)

*W; FRT42D Uba1^B2^*/*FRT42D* (Figure 1A, 1E red open squares)

*W; FRT42D Uba1^A1^*/*FRT42D* (Figure 1A, blue open squares)

*W; FRT42D Uba1^A3^*/*FRT42D* (Figure 1A, purple open squares)

*W; FRT42D Uba1^A5^*/*FRT42D* (Figure 1A, green open squares)

*W; FRT42D/+; elavgal4/+* (Figure 3C black open circles, 5E gray dashed line)^*^

*W; FRT42D Uba1^B1^*/*+* (Figure 5A, 5C black open squares)

*W; FRT42D Uba1^B1^/FRT42D Uba1^B1^* (Figure 3A, black bar, 3C, 3E, 5E, 5G, black open squares)

*W; FRT42D Uba1^B1^/FRT42D Uba1^B2^* (Figure 3A, gray bar)

*W; FRT42D Uba1^B1^*/*+; Ras^e1b^/+* (Figure 5A, 5C black filled-in squares)

*W; FRT42D Uba1^B1^*/*FRT42D Uba1^B1^; Ras^e1b^/+* (Figure 5E, 5G black-filled in squares)

^*^ *elavgal4* was present in these experimental controls as an additional control for parallel experiments using gal4/UAS-mediated transgene expression not included in this study. In multiple parallel experiments, *elavgal4* did not affect lifespan of these genotypes (data not shown).

### Immunohistochemistry

Adult brains were dissected, fixed in 4% paraformaldehyde, permeabilized in PBS-Tween, stained, and then imaged on a Leica TSC-SP confocal microscope. Primary antibodies were anti-βgal 40-1a (1∶10, DSHB); and anti-activated caspase 3 (1∶250, Promega). Secondary antibodies were Alexa-Fluor 488 goat anti-rabbit, Alexa-Fluor 488 goat anti-mouse, Molecular Probes/Invitrogen.

### Genetic Crosses

*Uba1^B1^* heterozygous flies with reduced gene dosage of Ras were generated by crossing *w; FRT42D Uba1^B1^/SM6-TM6B* to flies of the genotype *w; Ras^e1b^/TM6B. w; FRT42D Uba1^B1^/+*; *Ras^e1b^/+* flies were identified by the absence of the dominant visible markers *Cy, Hu, and Tb* found on the *SM6-TM6B* fused balancer, and the markers *Hu, and Tb* found on the *TM6B* chromosome. To generate homozygous *Uba1^B1^* flies with a reduced gene dosage of Ras, we crossed flies of the genetype *w; FRT42D Uba1^B1^/SM6-TM6B* to flies of the genotype *w; FRT42D Uba1^B1^*; *Ras^e1b^/SM6-TM6B*. *w; FRT42D Uba1^B1^/FRT42D Uba1^B1^; Ras^e1b^/+* flies were identified by the absence of the dominant visible markers *Cy, Hu, and Tb* found on the *SM6-TM6B* fused balancer chromosome.

Flies of the genotype *w; FRT42D Uba1^B1^/FRT42D* were generated by crossing *w; FRT42D Uba1^B1^/SM6-TM6B* to flies of the genotype *w; FRT42D*. *w; FRT42D Uba1^B1^/FRT42D* flies were identified by the absence of the dominant visible markers *Cy, Hu, and Tb* found on the *SM6-TM6B* fused balancer chromosome.

### Lifespan Assays

Flies of each genotype were collected within 24 hours of eclosion and placed in fresh vials and incubated at 25°C. Surviving flies were counted daily and transferred to fresh food every several days to prevent dessication of the food or growth of mold or bacteria. Data from flies collected on different days was pooled for each genotype.

### Climbing Assays

Climbing assays were performed similar to those described previously [55]–[56]. Age-matched flies of the indicated genotypes were placed into empty vials in small groups. When flies are tapped to the bottom of a vial, they immediately climb back to the top of the vial. To address motor function, flies were tapped to the bottom of the vial, and we counted the number of flies capable of climbing 4 centimeters in 5 seconds. Climbing assays were repeated five times for each group of flies at each time point. Due to the reduced survival of *Uba1^B1^*/*Uba1^B1^* and *Uba1^B1^*/*Uba1^B2^* flies, flies of these genotypes were collected each day; each small group was tested at age 5 days and 10 days, and the data pooled from the smaller groups.

### Statistical Analysis

Analysis of the climbing assays was performed using Microsoft excel spreadsheets. Lifespan averages, standard errors, and medians were calculated using Microsoft excel and Graphpad prism. Averages detailed in the text are lifespan ± s.e.m. Error bars in the graphs in Fig. 3A–B indicate standard deviation. Standard deviations for motor assays in Fig. 3A–B were calculated based on the deviation from five replicate tests of each population. Kaplan Meier survival analysis/comparison of overall survival curves using both Log-rank (Mantel-Cox) and Gehan-Breslow-Wilcoxon tests was performed using Graphpad Prism statistical software. P values are indicated in the text for each method, except in cases for which both gave P<0.0001 in which case only one P value is indicated.
